# The New World Language

**Published:** 1888-07

**Authors:** 


HALL'S
JOURNAL OF HEALTH

TRUTH DEMANDS NO SACRIFICE; ERROR CAN MAKE NONE.

VOL. 35. JULY, 1888 No 7.

### THE NEW WORLD LANGUAGE.

It is a most favorable sign of the times that the civilized nations of the world recognize the importance of a common language, the aquisition of which will enable the educated of whatever nationality to correspond and converse with one another. We published last month an account of the origin and systemization of Volapük, the new language. which is claimed to be very simple of construction and easily acquired. The foothold which it has already attained as a vehicle of business intercourse, among people widely apart in nationalty and dialect are very encouraging. In our present issue will be found a communication from a well known philologist, who having given a good deal of attention to this subject, points out some of the more glaring defects of Volapük, and suggests the need of a more euphoneus combination of vowel and consonant sounds, to express thoughts and emotions, which are in themselves pleasurable to the senses. However just this criticism may be, it is not strange that the author of the new language should have preserved the *umlant* of his native German, which renders their shades of pronunciation with their differencies of meaning difficult of acquision by the English student. This leaning towards the mother tongue would be likely to crop out in any similar work, by whomsoever undertaken, and in a measure lessen its general acceptability; but if as stated in Mr Whittaker's June article, it was essential for the Roman Catholic Priest Schleyer, to master fifty distinct languages, preliminary to his main task, it is not likely to be repeated by any considerable number of aspirants.

The system he presents as the result of years of labor, is only recommended for business and commercial international correspondeuce. If its harsh gutterals and ineuphoneous syllables debar it from the realm of poetry, love and song, or even the interchange of drawing room civilities, save in the absence of other modes of expression, it is not at all unlikely that it may yet like many an infantile language, undergo such changes and enlargements in a more general usage as to adapt it to all social needs, without adding unnecessary intricacies and complications.

Take for example the English, which is conceded to be the most comprehensive of living languages. In its earlier stages it was little more than a jargon, as Volapük is said to be now. Indeed the proficient English scholar of the present, would find it difficult to read and understand the English tongue as written prior to the Norman conquest, without the aid of an ample glossary, so great have been the changes in its construction since that period. All languages which have attained anything like completeness have been of slow growth, and too much should not be demanded of this latest vehicle of reciprocal intercommunication between distinct races. The object is one of great importance and ought to be appreciated alike by business men and scholars.

